# The Role of Environmental Processes and Geographic Distance in Regulating Local and Regionally Abundant and Rare Bacterioplankton in Lakes

**DOI:** 10.3389/fmicb.2021.793441

**Published:** 2022-02-16

**Authors:** John K. Pearman, Georgia Thomson-Laing, Jacob Thomson-Laing, Lucy Thompson, Sean Waters, Lizette Reyes, Jamie D. Howarth, Marcus J. Vandergoes, Susanna A. Wood

**Affiliations:** ^1^Coastal and Freshwater Group, Cawthron Institute, Nelson, New Zealand; ^2^GNS Science, Lower Hutt, New Zealand; ^3^School of Geography, Environment and Earth Sciences, University of Victoria, Wellington, New Zealand

**Keywords:** 16S rRNA gene metabarcoding, bacterioplankton, rare bacteria, abundant bacteria, environmental processes, geographic distance

## Abstract

Bacteria are vital components of lake systems, driving a variety of biogeochemical cycles and ecosystem services. Bacterial communities have been shown to have a skewed distribution with a few abundant species and a large number of rare species. The contribution of environmental processes or geographic distance in structuring these components is uncertain. The discrete nature of lakes provides an ideal test case to investigate microbial biogeographical patterns. In the present study, we used 16S rRNA gene metabarcoding to examine the distribution patterns on local and regional scales of abundant and rare planktonic bacteria across 167 New Zealand lakes covering broad environmental gradients. Only a few amplicon sequence variants (ASVs) were abundant with a higher proportion of rare ASVs. The proportion of locally abundant ASVs was negatively correlated with the percentage of high productivity grassland in the catchment and positively with altitude. Regionally rare ASVs had a restricted distribution and were only found in one or a few lakes. In general, regionally abundant ASVs had higher occupancy rates, although there were some with restricted occupancy. Environmental processes made a higher contribution to structuring the regionally abundant community, while geographic distances were more important for regionally rare ASVs. A better understanding of the processes structuring the abundance and distribution of bacterial communities within lakes will assist in understand microbial biogeography and in predicting how these communities might shift with environmental change.

## Introduction

Many ecological studies have overarching goals to understand the biodiversity and spatial distribution (biogeography) of organisms and to determine the drivers underlying these patterns. Microbes are ubiquitous and amongst the most abundant organisms on Earth, playing vital roles in biogeochemical and metabolic processes (Whitman et al., 1998; Bardgett et al., 2008). The discrete nature of lakes and their diverse environmental conditions provides an ideal opportunity to investigate large scale biogeographical patterns of microbial communities.

It has been postulated that local environmental processes and geographic distances (dispersal related) factors are potential mechanisms in structuring microbial biogeography (Liu et al., 2019; Sadeghi et al., 2021). Environmental processes include abiotic (e.g., nutrients) and biotic (e.g., predation and competition) factors that regulate the community structure (Zhou and Ning, 2017). Microbial communities have previously been shown to be affected by within lake conditions (e.g., pH, nutrient concentrations, and temperature; Hoostal and Bouzat, 2008; Ruuskanen et al., 2018; Pearman et al., 2020), as well as catchment properties, such as land use (Kraemer et al., 2020) and altitude (Hayden and Beman, 2016; Li et al., 2017; Pearman et al., 2020). Conversely, neutral processes suggest that all taxa are functionally equivalent and not strongly affected by environmental effects and that community assembly is thus driven by variations in dispersal coupled with ecological drift (Hubbell, 2001; Barberán et al., 2014a; Zhou and Ning, 2017). The relative importance of environmental processes and dispersal related processes on structuring bacterial communities in lake systems is conflicting with evidence of environmental selection in some studies (Beisner et al., 2006; Gucht et al., 2007), while others suggest dispersal related processes are the most important factor (Sloan et al., 2006; Drakare and Liess, 2010). There is also compelling research which suggests that neither process is dominant in regulating microbial distribution patterns in lakes (Langenheder and Ragnarsson, 2007; Langenheder and Székely, 2011; Liu et al., 2015).

With the development of molecular techniques such as high throughput sequencing, the composition of microbial communities is increasingly being investigated. A common feature in microbial biospheres is a skewed distribution of species abundance with communities composed of a small number of species that are highly abundant, and a large number of rare species (Sogin et al., 2006; Nemergut et al., 2011; Pedrós-Alió, 2012; Logares et al., 2014; Lynch and Neufeld, 2015; Jousset et al., 2017) which is reminiscent of the classical ecological patterns observed for plants and animals (Magurran and McGill, 2011). This skewed distribution has been shown to be true at both local and regional scales. Logares et al. (2014) define locally abundant taxa as those with a relative abundance >1% in a sample and locally rare as <0.01%. On a regional scale, abundant taxa are defined as having a relative abundance across all samples of >0.1% with regionally rare taxa having an average of <0.001%. There is increasing evidence that these components of bacterial communities respond differently to environmental processes and dispersal related processes (Lindström and Langenheder, 2012). Regionally abundant taxa often have high dispersal rates, broad fitness, and the ability to proliferate across a wide range of environmental conditions and have a high occupancy rate (Barberán et al., 2014b). In contrast, locally rare species, due to their low abundance, tend to have limited dispersal capabilities and thus a restricted distribution and are subsequently also regionally rare. However, rare species often undertake important functions in the community which are disproportionate to their abundance (Pester et al., 2010; Campbell et al., 2011; Mouillot et al., 2013). Rare species may provide functional resilience by being dormant/inactive and acting as a genetic reservoir until environmental conditions become favorable for their growth at which point these regionally rare species can become locally abundant (Jones and Lennon, 2010; Lennon and Jones, 2011; Caporaso et al., 2012; Shade et al., 2014; Shade and Gilbert, 2015).

The biogeography of regionally abundant and rare microbial taxa in lakes has been investigated with differing results. Bai et al. (2020) found that among interconnected lakes dispersal related processes were more prominent in structuring both the abundant and rare communities. For more isolated lakes, environmental processes had a higher contribution in structuring the abundant compared to the rare community which were dispersal limited (Li et al., 2017). An assessment of lakes and reservoirs in China showed the opposite trend with dispersal related processes being more important in structuring the abundant community (Liu et al., 2015). To enhance knowledge on the factors regulating the composition of abundant and rare bacteria in lakes, more wide-scale studies of microbial communities across geographic distance and environmental gradients are required. This will assist in understanding factors that structure microbial communities and the potential resilience of these organisms to environmental change.

In this study, we used DNA metabarcoding to explore the distribution of planktonic bacteria in lake surface waters, termed bacterioplankton, at a national scale in 167 New Zealand lakes encompassing broad environmental gradients. We hypothesized that (i) there would be few abundant Amplicon Sequence Variants (ASVs) with rare ASVs accounting for the majority of the observed richness on both a local and regional scale; (ii) environmental variables would be different for structuring the composition of locally abundant and rare ASVs; (iii) the taxonomic makeup of regionally abundant and rare ASVs would differ; (iv) regionally abundant ASVs would have high occupancy across the lakes with rare ASVs having a restricted occupancy; and (v) environmental processes would be strong drivers of the composition of regionally abundant communities, while geographic distance would contribute more in rare communities.

## Materials and Methods

### Study Lakes

**FIGURE 1:**
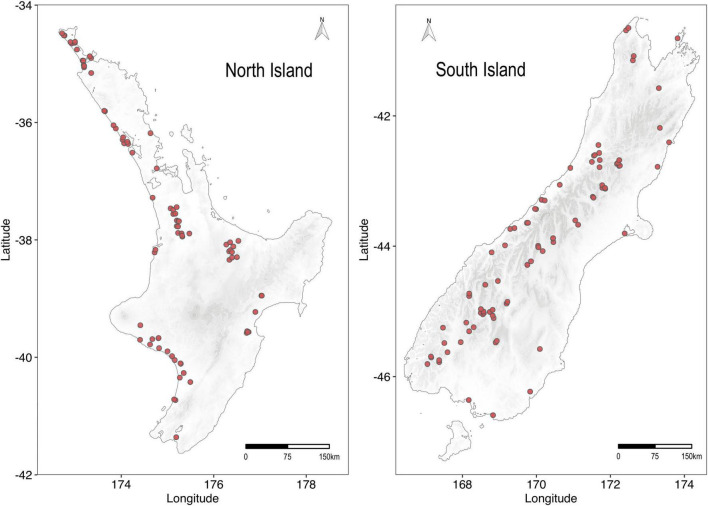
Map showing location of study lakes. Co-ordinates for the lakes are given in Supplementary Table 1.

A total of 167 lakes, which were not hydrologically connected, were sampled across New Zealand between 23 January 2019 and 15 February 2021 (Figure 1 and Supplementary Table 1). Lakes ranged in altitude from 2.6 m (Lake Forsyth) to 1,839 m (Duncan Stream Tarn) above sea level, in depth from 0.9 m (Lake Runanga) to 126 m (Lake Rotoiti) and lake area from 1 (Lake Kawau) to 17,273 ha (Lake Pukaki).

Altitude and lake depth were assessed *in situ* at each lake. Satellite imagery available in the Land Cover Database Version 5 (LCDBv5, Landcare Research New Zealand Ltd., Lincoln, New Zealand)^1^ was used to derive seven broad land cover variables: (1) Native vegetation, (2) Urban, (3) Non-native vegetation, (4) Forestry, (5) High Production Grassland (HPG; proportion of high productivity grassland (exotic grasslands rotationally grazed for wool, lamb, beef, or dairy), (6) Low Production Grassland (LPG; agricultural grassland with a low stock density), and (7) Other. Specific land cover variables for each category are detailed in Supplementary Table 1. The percentages of the catchment attributed to each of these land cover variables were used as environmental variables.

### Water Quality Sampling and Analysis

Prior to sampling, a side scan sonar survey (Lowrance, Tulsa, United States) or depth sounder (Norcross HawkEye H22PX, Orlando, United States) was used to find the deepest part of the lake. Subsequent sampling was undertaken at this site except for lakes deeper than 100 m where sampling was undertaken in shallower bays (Supplementary Table 1). Secchi depth was recorded at all lakes, and water column profiles for temperature (°C) and dissolved oxygen (DO% and mg/L) were measured using a RBRmaestro^3^ Multi-Channel Logger (RBR, Ottawa, Canada).

Surface water (1 L) for chlorophyll *a* was collected at the deepest point in the lake and kept on ice until further processing. A subsample (up to 600 mL) was filtered (GF/C, Whatman, Maidstone, United Kingdom), and the volume filtered was recorded. Filters were placed in aluminum foil and stored in the dark (−20^°^C). Chlorophyll *a* analysis was undertaken following the APHA 10200 H method at Watercare Laboratories (Auckland, New Zealand) with a reporting limit of 0.0006 mg L^–1^.

Water samples (1 L) were collected using an integrated tube sampler through the surface mixed layer as determined by the RBR profile. Subsamples (40 mL) were filtered (low bleed 0.45 μm) for dissolved organic carbon (DOC; Supplementary Table 1). Analysis of DOC was undertaken by combustion analysis at 850°C using APHA 5310 B methods with reporting limits of 0.5 g/m^3^ (Supplementary Table 1).

### Bacterioplankton Sampling

Surface water samples (500 mL) were collected in triplicate. For each triplicate, a subsample (up to 100 mL) of water was filtered (S-Pak filter, 0.22 μm pore size, 4.7 cm dia., Millipore Sigma, Burlington, United States). The volume filtered was recorded, and filters were stored in sterile tubes (1.5 mL) at −20°C until DNA extraction. All filtering apparatus were bleached between lakes.

### DNA Extraction, Polymerase Chain Reaction, High Throughput Sequencing, and Bioinformatics

Molecular analyses [i.e., DNA extraction, Polymerase Chain Reaction (PCR) set-up, template addition, and PCR analysis] were conducted in dedicated sterile separate laboratories, with sequential workflow to reduce potential cross-contamination. UV sterilization was undertaken for 15 min before and after each procedure in the DNA extraction, amplification set-up, and template addition laboratory. The PCR set-up and template addition were undertaken in laminar flow cabinets with HEPA filtration. Aerosol barrier tips (Eppendorf, Hamburg, Germany or Axygen, Corning, United States) were used throughout.

Bacterial filters were cut into five pieces using sterile scissors in a laminar flow cabinet and placed into the power bead tube of a DNeasy PowerSoil Kit (Qiagen, Hilden, Germany). DNA was extracted following the manufacturer’s instructions on a QIAcube sample preparation robot (Qiagen). A negative extraction control was included every 23 samples.

The V3-V4 regions of the bacterial 16S rRNA gene were amplified using the primers: 341F: 5’-CCT ACG GGN GGC WGC AG-3’ and 805R: 5’-GAC TAC HVG GGT ATC TAA TCC-3’ (Klindworth et al., 2013). The primers were modified with the Illumina™ overhang adapters to allow dual indexing as described by Kozich et al. (2013). Amplification was undertaken *via* PCR in 20 μL volumes performed in triplicate as described by Pearman et al. (2020) with negative controls included. PCR triplicates were combined, and 25 μL of pooled product was placed in SequalPrep™ Normalization plates (Thermo Fisher Scientific, Waltham, United States) for cleaning and normalization. The cleaned product had a DNA concentration of ∼1 ng mL^–1^ which was sequenced on an Illumina Miseq™ platform at the Auckland Genomics Facility. Library preparation was undertaken following the Illumina 16S metagenomics library prep manual. The only exception was that after the indexing PCR, 5 μL of each sample was pooled, and a single clean-up was undertaken on the pool instead of samples being individually cleaned. The library contained extraction, PCR, and sequencing controls. A bioanalyzer was used to assess library pool quality and concentration. The library was diluted to 4 nM, denatured, and diluted to a final loading concentration of 7 ρM with a 15% PhiX spike. Raw reads are stored in the NCBI short read archive under the accession: PRJNA762383.

### Bioinformatics

Multiple sequencing runs were undertaken with the sequencing runs being processed separately before being combined after chimera detection. Sequences were processed bioinformatically by first removing primer sequences from the raw reads using cutadapt (1 mismatch allowed; Martin, 2011). Subsequent to primer removal, sequences were processed using the *DADA2* package (Callahan et al., 2016) within R (R Core Team, 2020). Briefly, reads were truncated (230 and 228 bp for forward and reverse reads, respectively) and filtered with a maximum number of “expected errors” (maxEE) threshold of four (forward reads) and six (reverse reads). Reads with higher maxEE were discarded from further analysis. The first 10^8^ bp of sequences were used to construct a parametric error matrix which was used to infer amplicon sequence variants (ASVs) after dereplication. Singletons were removed, and remaining paired-end reads were merged with a maximum mismatch of 1 bp and a required minimum overlap of 10 bp. Chimeric sequences were detected and removed using removeBimeraDenovo within the *DADA2* package. The multiple sequencing runs were combined together before taxonomy was undertaken on the whole dataset. Taxonomy was assigned against the SILVA 138 reference database (Pruesse et al., 2007) using the RDP classifier (Wang et al., 2007) with a bootstrap of 70 to allow classifications at higher taxonomic levels. The ASV table, taxonomy, and metadata were combined into a *phyloseq* object (McMurdie and Holmes, 2013), and sequences assigned as eukaryotes, chloroplasts, and mitochondria were removed. To minimize issues of contamination, the negative controls (extraction, PCR, and sequencing) were investigated. Of the 31 negative controls assessed, only two had >3,000 reads with 24 having <1,000 reads. To remove contaminants, each sequencing run was processed separately. The maximum number of reads for each ASV found in the negative controls of the sequencing run was removed *via* subtraction from the corresponding samples (Bell et al., 2019). Any ASV in the samples that had fewer reads than in the controls was presumed to be a contaminant and removed from that sample (read abundance = 0). To remove potentially erroneous ASVs, those ASVs that were not present in at least two replicates within a lake were removed from that lake. To enable comparisons amongst lakes, triplicate samples were merged and subsampled, without replacement to an even depth of 35,000 reads.

### Statistical Analysis

There were 16 measured environmental variables (catchment characteristics and physiochemical variables) available for this dataset (Supplementary Table 1). These were tested for co-linearity, and one of co-linear variables was selected. This reduced the dataset to 15 variables (latitude, altitude, lake area, proportion of high productivity grassland in the catchment, proportion of low productivity grassland in the catchment, proportion of native vegetation in the catchment, proportion of non-native in the catchment, proportion of forestry in the catchment, Secchi disk depth, surface water temperature, surface dissolved oxygen, concentration of chlorophyll *a* and concentration of dissolved organic carbon, max. depth, and sampling season). These variables were scaled and centered using the R package *caret* (Kuhn, 2008). Missing values were imputed *via* bagging which fits a bagged tree model for each predictor (as a function of all others) in *caret*.

The number of observed ASVs per lake was calculated, and a Kruskal-Wallis test was used to explore differences in the number of ASVs based between the North and South Islands of New Zealand.

#### Local Analysis

Locally abundant ASVs were classified as having a relative abundance within a lake of >1%, while locally rare ASVs had a relative abundance of <0.01% when present in the lake.

Generalized Linear Models (GLMs) were used to assess the impact of environmental variables on total community richness and proportion of reads of locally abundant or rare ASVs in a lake using the R package *MASS* (Venables and Ripley, 2002). A step wise (both directions) process was used to determine the best model based on the lowest Akaike Information Criterion (AIC).

Distance Based Redundancy Analysis (dbRDA; McArdle and Anderson, 2001) was used to investigate the relationship between the locally abundant bacterioplankton communities grouped at the genus level and the lake’s environmental characteristics. The dbRDA was not undertaken on the rare component due to the restricted distribution of these ASVs which prevents any meaningful relationships being identified. The dbRDA was based on the Bray-Curtis dissimilarity measure undertaken on the bacterial composition data amalgamated at the genus level of classification and undertaken in R using the *phyloseq* package.

#### Regional Analysis

To assess abundance patterns on a regional scale, ASVs were classified into three groups based on average relative abundance across all 167 lakes (criteria from Logares et al., 2014): regionally abundant (>0.1%), regionally intermediate (>0.001% and <0.1%), and regionally rare (<0.001%). Differences in the relative abundance of taxonomic classes in both the regionally abundant and rare categories was statistically tested with a paired Wilcox test within R using a two-sided alternative hypothesis.

To analyze the relative contributions of environmental and geographical distance to the bacterioplankton, Mantel and partial Mantel correlations (method = Pearson) were calculated between the three ASV Bray Curtis dissimilarity matrices (total, regionally abundant, and regionally rare) and environmental and geographical distances (Legendre and Legendre, 2012) within the *vegan* package in R (Oksanen et al., 2007). Geographic distances (haversine distances) were calculated from sampling coordinates with the function distHaversine in the R package *geosphere* (Hijmans et al., 2017). Fourteen out of the 15 environmental variables (the categorical factor “sampling season” was excluded) were used to calculate a Euclidean distance matrix.

## Results

### Overall Diversity and Explanatory Variables

In total, there were 14,763 ASVs across the 167 lakes after rarefaction and removal of ASVs that were not in at least two replicates. When the data from all lakes were combined, bacterioplankton were predominantly comprised of Gammaproteobacteria (mean 20.9 ± 12.3%), Actinobacteria (20.9 ± 10.8%), Bacteroidia (14.8 ± 8.8%), Alphaproteobacteria (12.3 ± 7.9%), and Verrucomicrobiae (9.7 ± 6.4%) (Supplementary Figure 2 and Supplementary Table 2). Cyanobacteria and Acidimicrobiia, while not present in all lakes, contributed substantially (8.0 ± 10.5% and 5.7 ± 4.3%, respectively).

On average, 333 ASVs (range 85–1,108 ASVs) were identified within each lake. There was a significant difference in the number of ASVs per lake between the North and South Islands (Kruskal-Wallis: chi-squared = 12.9, *p* < 0.0003), with the South Island having a lower richness than the North Island (mean 288 ± 143 compared to 381 ± 218 ASVs).

### Locally Abundant and Rare Communities

In total, there were 1,156 ASVs (7.8% of the community) that had a relative abundance of >1% within a lake (locally abundant). There were on average 21.4 ± 4.6 locally abundant ASVs per lake. There were 6,567 ASVs (44.5%) that had a relative abundance when present of <0.01% within a lake (locally rare). There were on average 65.3 ± 80.7 locally rare ASVs per lake. Locally abundant ASVs accounted for 63.9 ± 11.2% of the reads within a lake while the locally rare ASVs accounted for 0.36 ± 0.42% of reads (Supplementary Figure 3).

Generalized linear models showed that the proportion of reads accounted for by locally abundant ASVs had a significantly negative relationship with high productivity grassland (*p* = 0.012) and lake depth (*p* < 0.001), while a significant positive partial effect was observed for Secchi disk depth (*p* < 0.001) and temperature (*p* < 0.042; Supplementary Figure 4A). For the locally rare ASVs, five factors (dissolved organic carbon, Secchi disk depth, proportion of native and low production grassland in the catchment, and dissolved oxygen) had significant negative effects on the proportion of reads accounted for by locally rare ASVs (Supplementary Figure 4B).

**FIGURE 2:**
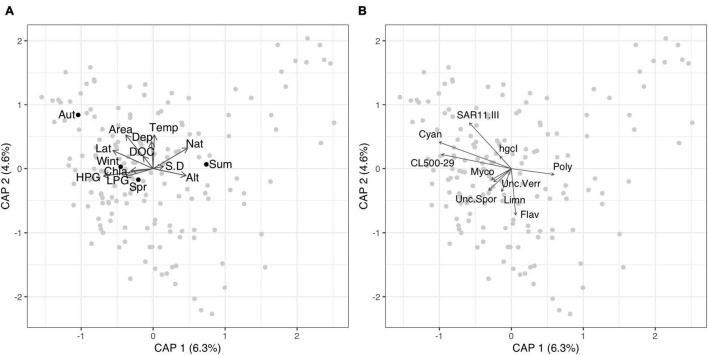
Distance based redundancy analysis of the locally abundant taxa at the genus level. The gray points represent each lake. The black dots represent the centroids of the categorical variable season. Vector arrows shows the continuous environmental variables in panel **(A)** and the top 10 genera of the locally abundant community **(B)**. Taxa codes are: Flav, Flavobacteriaceae; hgcI, clade hgcI of the family Sporichthyaceae; Cyan, Cyanobium; SAR11.III, Clade III of the order SAR11; Unc.Spor, Unclassified family Sporichthyaceae; CL500-29, Clade CL500-29 of the family Ilumatobacteraceae; Limn, Limnohabitans; Poly, Polynucleobacter; Unc.Verr, Unclassified class Verrucomicrobiae; Myco, Mycobacterium. Environmental variables codes are: DOC, dissolved organic carbon; Temp, Surface water temperature; HPG, high producing grassland; LPG, low producing grass; Nat, native catchment; Alt, altitude; Lat, Latitude; S.D, Secchi disk depth; Depth, Max. depth; Wint, Winter sampling (June–August); Spr, Spring sampling (September–November); Sum, Summer sampling (December–February); Aut, Autumn sampling (March–May).

Distance based redundancy analysis of locally abundant taxa at the genus level showed that the environmental variables assessed only explained a low percentage of the variation (6.3 and 4.6% on axis 1 and 2, respectively) (Figure 2 and Supplementary Table 3). The genus *Cyanobium* was positively associated with latitude. Clade III of the class SAR11 was positively with dissolved organic carbon and lake area. Flavobacterium was negatively correlated with temperature, while Polynucleobacter was positively associated with altitude.

### Regionally Abundant, Intermediate, and Rare Taxa Comparison

**FIGURE 3:**
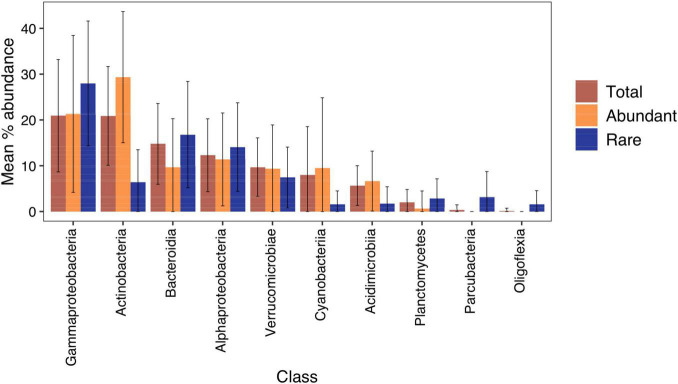
Mean percentage abundance of bacterial classes in 167 New Zealand lakes. Error bars show one standard deviation. Classes with <0.1% mean abundance were not plotted. Regionally abundant = average relative abundance of >0.1%, and rare <0.001%. Percentage abundances for each lake are in Supplementary Figure 2.

There were significant differences in classes that made up the regionally abundant and rare fraction of the community (Figure 3). In total, there were 13 classes represented in the abundant component with 113 in the rare. Actinobacteria, Cyanobacteria, and Acidimicrobiia accounted for a significantly higher proportion of reads (paired Wilcox test: *p* < 0.001) in the abundant fraction, while Gammaproteobacteria, Alphaproteobacteria, Planctomycetes, and Parcubacteria were more prominent in the rare fraction (paired Wilcox test: *p* < 0.001).

**FIGURE 4:**
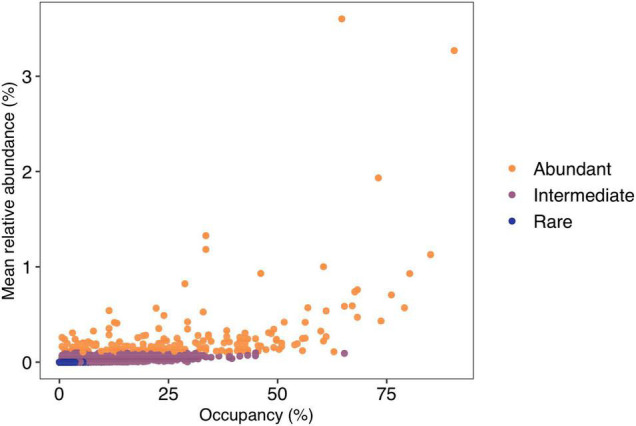
Occupancy rate (number of lakes) for amplicon sequence variants vs. mean relative abundance (%). Lakes are colored by their abundance category. Regionally abundant = average relative abundance of >0.1%, intermediate <0.1% but >0.001%, and rare <0.001%.

No ASVs were shared amongst all lakes, with only five ASVs (0.03%) found in more than 75% of lakes. These ASVs belonged to Sporichthyaceae (Actinobacteria = three ASVs), Methylophilaceae (Gammaproteobacteria), and Comamonadaceae (Gammaproteobacteria). The majority (14,188; 96.1%) of ASVs were found in <10% of lakes, with 7,903 ASVs (52.5%) found only in a single lake. There were 191 regionally abundant ASVs and 10,212 rare ASVs. The remaining 4,377 ASVs were classified as intermediate (Figure 4).

Occupancy of regionally rare ASVs was restricted with no rare ASVs found in more than 10% of lakes. For the regionally intermediate ASVs, the majority were found in <50% of the lakes, with only one ASV found in more than 50% of lakes. Of the regionally abundant ASVs, 16% (30 ASVs) were present in more than half the sites with a maximum occupancy of ∼90% of lakes. These 30 ASVs represented 5 classes and 12 families. The most frequent taxonomic classification of these widely distributed abundant ASVs belonged to the family Sporichthyaceae (9 ASVs; Actinobacteria) with a further 5 ASVs belonging to the family Comamonadaceae. However, there were also abundant ASVs that had restricted occupancies, with ∼10% (18 ASVs) of abundant ASVs having an occupancy of <5% of lakes. These ASVs belonged to 12 families within 7 classes.

### Environmental and Geographic Distance Relationships for Total and Regionally Abundant and Rare Amplicon Sequence Variants

Mantel and partial Mantel tests using all ASVs showed that environmental processes had a stronger relationship with total bacterioplankton community composition compared to geographic distance especially when using the partial mantel tests (Table 1). A similar pattern was observed for the regionally abundant fraction of the community. However, the regionally rare fraction of the community showed a different trend with geographic distance and environmental processes having similar effects (Table 1).

**TABLE 1:** Mantel and partial Mantel tests comparing the correlation between community composition and either environmental or geographic distance matrices for the total community and the regionally abundant and rare communities.

	Mantel		Partial mantel	
	Environmental	Geographic	Environmental	Geographic
Total	0.268	0.170	0.234	0.105
Abundant	0.226	0.173	0.192	0.106
Rare	0.191	0.185	0.149	0.142

*All p < 0.001.*

## Discussion

The bacterioplankton community of New Zealand’s lakes are dominated by Gammaproteobacteria and Actinobacteria, a pattern that has been observed in other lake systems globally (Hugoni et al., 2017; Jiao et al., 2018; Kiersztyn et al., 2019; Bai et al., 2020).

### Local Communities

As we hypothesized and in agreement with other microbial studies, there was a skewed distribution to the bacterioplankton community with a low number of locally abundant ASVs and a higher proportion of rare ASVs (Logares et al., 2014). The measured environmental factors only explained a small proportion of the variability in the bacterioplankton community. There are multiple plausible reasons for this. Other environmental variables (e.g., nutrients and pH) that were not investigated and so were not accounted for in the models could explain some of the variation. Biological impacts such as grazing and virus infection were also not taken into account but have previously been shown to have impacts on bacterioplankton communities (Tijdens et al., 2008; Shurin et al., 2012; Li et al., 2020). Finally, the lakes in the study crossed multiple different environmental, chemical, and physical gradients and the interaction of these gradients may confound results.

Despite the low proportion of variation in community composition explained, some associations were present between the measured environmental variables and locally abundant ASVs aggregated at the genus level. The picocyanobacterial genus *Cyanobium* showed an association with lower latitudes and with increasing amounts of dissolved organic carbon which is often related to lake eutrophication, typical of lowland lakes in parts of the North Island. Previous work by Wood et al. (2017) has shown that a single cyanobacterial taxon can dominate in eutrophic lakes. This pattern was also observed in the present study, with individual ASVs sometimes dominating the cyanobacterial proportion of the community. These ASVs included taxa from the picocyanobacterial family Cyanobiaceae, and the Microcystaceae and Phormidiaceae which contain many bloom forming taxa.

The locally abundant ASVs accounted for a lower proportion of the community in shallow lakes with a higher percentage of high productivity grassland in the catchment with low Secchi disk depths. In contrast, the proportion of reads accounted for by rare ASVs increased in lakes with low Secchi disk depths, which can be typical of nutrient enriched lakes. There was also a positive association of high productivity grassland in the catchment, which is often related to lake nutrient enrichment due to fertilizer use, with the overall ASV richness in a lake. Previous studies have shown that a higher diversity is observed in more eutrophic lakes (Jankowski et al., 2014; Kiersztyn et al., 2019) and have attributed it to the release from nutrient limitations in these high productivity lakes allowing more species to co-exist.

### Regional Diversity Patterns

Microbial communities in a variety of habitats, including lakes, have been shown to have few regionally abundant taxa with most diversity being rare and of low relative abundance (Pedrós-Alió, 2012; Liu et al., 2015; Jiao et al., 2018; Bai et al., 2020). The bacterioplankton of New Zealand’s lakes showed a similar pattern with ∼1% of ASVs abundant regionally and ∼70% of ASVs classed as regionally rare.

The taxonomic composition of regionally abundant and rare communities varied, as has been previously shown in other lake systems (Bai et al., 2020). The phylogenetic diversity of the abundant ASVs was far more restricted with only a small number of classes. This may be because abundant ASVs belong to taxa which can proliferate in lake environments, while the regionally rare component of the community can also include transient ASVs from other habitats (e.g., terrestrial taxa) which are imported into the lake system but are unable to grow/propagate in aquatic environments (Newton et al., 2011).

Distribution patterns also differ between regionally abundant and rare components of the bacterioplankton. Rare ASVs had a restricted occupancy with the abundant ASVs, in general, having a broader distribution within lakes. These results are similar to other studies focusing on lake bacteria (Liu et al., 2015; Jiao et al., 2018; Bai et al., 2020), although they differ from the cosmopolitan distribution suggested by Pedrós-Alió (2012). One of the reasons proposed for the wide occupancy of abundant taxa is their ability to proliferate across a broad range of environmental conditions (Barberán et al., 2014b). Actinobacteria had the highest number of ASVs found in more than 50% of the lakes. Actinobacteria and especially members of the hgcI clade, within the family Sporichthyaceae, have been shown to have high abundance and frequency of occupancy in a variety of freshwater environments (Glöckner et al., 2000; Warnecke et al., 2004; Newton et al., 2011; Liu et al., 2015; Jiao et al., 2018; Kraemer et al., 2020). Recent genomic analysis of freshwater Actinobacteria has revealed their ability to degrade complex organic materials as well as having high affinity broad uptake systems, which when combined with a high surface area to volume ratio makes them competitive in nutrient deplete environments (Ghai et al., 2014). Further, Actinobacteria have been shown to have a high degree of inter- and intra-specific variation in their metabolic pathways (Neuenschwander et al., 2018). Their small size and cell wall structure may also protect them from protistan grazing (Allgaier and Grossart, 2006) which could also explain the ability of members of Actinobacteria to reach high abundances in lake systems.

Taxa that were present in the regionally abundant component did not all have high occupancy. For example, Cyanobacteria were predominantly in the regionally abundant component but had a more restricted distribution with no Cyanobacteria ASVs present in more than 50% of the lakes. This restricted distribution but high average abundance may suggest that cyanobacteria could be classified as conditionally rare taxa (Shade et al., 2014). These taxa are normally rare but occasionally become highly abundant when environmental conditions permit. Indeed, cyanobacteria are known to be able to remain dormant in unfavorable conditions with periodical increases in abundance and the formation of blooms within freshwater systems when conditions are preferable (Reinl et al., 2021). Previous studies investigating freshwater phytoplankton, including cyanobacteria, have shown that local environmental processes predominantly drive biodiversity patterns with spatial patterns contributing to a lesser extent (Stomp et al., 2011; Touzet et al., 2013; Wood et al., 2017). The predominance of environmental processes over geographic distance may explain the distribution patterns of taxa with high abundance yet restricted occupancy.

Gammaproteobacteria were major components of both the abundant and rare community, although they had a significantly higher contribution to the rare component. It has previously been suggested that the wide diversity of Gammaproteobacteria taxa observed in lake systems could indicate that they include transient members of the community brought in from the surrounding environment or common taxa which are always at low abundance (Newton et al., 2011). ASVs attributed to the families Comamonadaceae and Methylophilaceae, now classified within Gammaproteobacteria, had some of the highest lake occupancy. Members of the family Methylophilaceae have been shown to play a key role in the carbon cycle of aquatic ecosystems, with the pelagic members showing genome streamlining (Salcher et al., 2019). The members of Comamonadaceae that were most frequently observed belonged to the genus *Limnohabitans* which are often abundant in freshwater environments (Willems, 2014). This genus has both generalist and specialist lineages (Jezberová et al., 2017), in agreement with the current results with ASVs related to this genus frequent in both the abundant and rare component of the community.

Our results indicated that the regionally abundant and rare components of the bacterial community were structured differently with environmental processes having a greater impact on the abundant community, while geographic distance and environmental processes contributed similarly for the rare component. The restricted distribution of the rare taxa indicates that processes such as dispersal limitation may contribute substantially to the structuring of these communities. This is further supported by our partial mantel test results which showed geographic distances made an equal contribution to structuring the rare community compared with the environmental processes. Lakes can be considered as aquatic islands surrounded by terrestrial habitats. Wilkinson et al. (2012) showed that aerial dispersal was feasible for bacteria with waterbirds another possible dispersal vector given they have been shown to move zooplankton and dinoflagellates between lakes (Figuerola et al., 2005; Tesson et al., 2018). However, the low abundance of rare taxa within a lake could limit the probability of dispersal amongst lakes. In contrast, the abundant component of the bacterial community would have an increased probability of dispersal amongst lakes and abundances within a lake are subsequently determined by local environmental processes. This assumption is supported by the partial mantel test results and is in agreement with a study on Chinese lakes (Bai et al., 2020).

The logistical constraints of sampling across the length and breadth of New Zealand meant that lakes were sampled in different seasons and years. Seasonal differences have previously been shown in planktonic communities (Loewen et al., 2020; Schallenberg et al., 2021) with the prevalence of local and regional level drivers changing (Loewen et al., 2020). However, we acknowledge that the sampling design meant that groups of lakes close together were sampled at the same period of time which could impact the assessment of the comparison between environmental processes and geographic distance. Where possible, future studies should attempt to limit sampling of bacterioplankton to a single season.

## Conclusion

In agreement with our first hypothesis, there was a skewed distribution to bacterioplankton communities with more rare ASVs than abundant ASVs. Only a small amount of the variation in the locally abundant community could be attributed to the environmental variables, suggesting further work is required to identify drivers of abundant bacterioplankton across large spatial scales. The composition of the abundant and rare components was substantially different with abundant taxa having a more restricted phylogenetic diversity. We showed that regionally abundant ASVs in general had a higher lake occupancy than regionally rare ASVs, although some regionally abundant taxa were restricted to a small number of lakes. The regionally abundant component had a higher contribution from environmental processes compared to geographic distance, while for the rare fraction geographic distance was more important and contributed equally with environmental processes.

## Data Availability Statement

The datasets presented in this study can be found in online repositories. The names of the repository/repositories and accession number(s) can be found below: https://www.ncbi.nlm.nih.gov/bioproject/PRJNA762383/.

## Author Contributions

JP, SAW, JH, and MV contributed to the conception and design of the study. GT-L, JT-L, LT, LR, and SW undertook the laboratory work. JP undertook the analysis of the data. JP and SAW wrote the manuscript. All authors helped with the field sampling and involved in revision and approval of the manuscript.

## Conflict of Interest

The authors declare that the research was conducted in the absence of any commercial or financial relationships that could be construed as a potential conflict of interest.

## Publisher’s Note

All claims expressed in this article are solely those of the authors and do not necessarily represent those of their affiliated organizations, or those of the publisher, the editors and the reviewers. Any product that may be evaluated in this article, or claim that may be made by its manufacturer, is not guaranteed or endorsed by the publisher.

## Footnotes

1
https://lris.scinfo.org.nz/layer/104400-lcdb-v50-land-cover-database-version-50-mainland-new-zealand/

## Supplementary Material

Supplementary Figure 1 Selected environmental and physico-chemical variables and dominant land uses at the study lakes. The environmental data, physico-chemical results, and land use data for all sites are provided in Supplementary Table 1.

Supplementary Figure 2 Percentage abundance of bacterial classes in 174 New Zealand lakes. Only classes that comprised >0.1% of community are shown.

Supplementary Figure 3 Violin plots showing **(A)** the richness of locally abundant and rare ASVs in a lake and **(B)** the percentage of reads accounted for by the locally abundant and rare ASVs per lake.

Supplementary Figure 4 Partial effects for the Generalized Linear Model (GLM) for; **(A)** the proportion of reads attributed to locally abundant amplicon sequence variants (ASVs; >1% of ASVs within a lake) were negatively affected by HPG (*p* = 0.012) and depth (*p* < 0.001) while being positively affected by S.D (*p* < 0.001) and altitude (*p* = 0.030). The relationship with Temp and LPG was not significant. **(B)** The proportion of reads attributed to the locally rare ASVs (<0.01% within a lake) with all included environmental variables being negatively associated (*p* < 0.01). HPG, percentage of highly productive grassland in the catchment; LPG, percentage of low productivity grassland in the catchment; Temp, surface water temperature; S.D, Secchi disk depth; DOC, dissolved organic carbon; DO, dissolved oxygen.
