# Supplementary figures and images for: The Role of Environmental Processes and Geographic Distance in Regulating Local and Regionally Abundant and Rare Bacterioplankton in Lakes
Source: Front Microbiol. 2022 Feb 16;12:793441. doi: 10.3389/fmicb.2021.793441 (PMC8888906; doi:10.3389/fmicb.2021.793441)

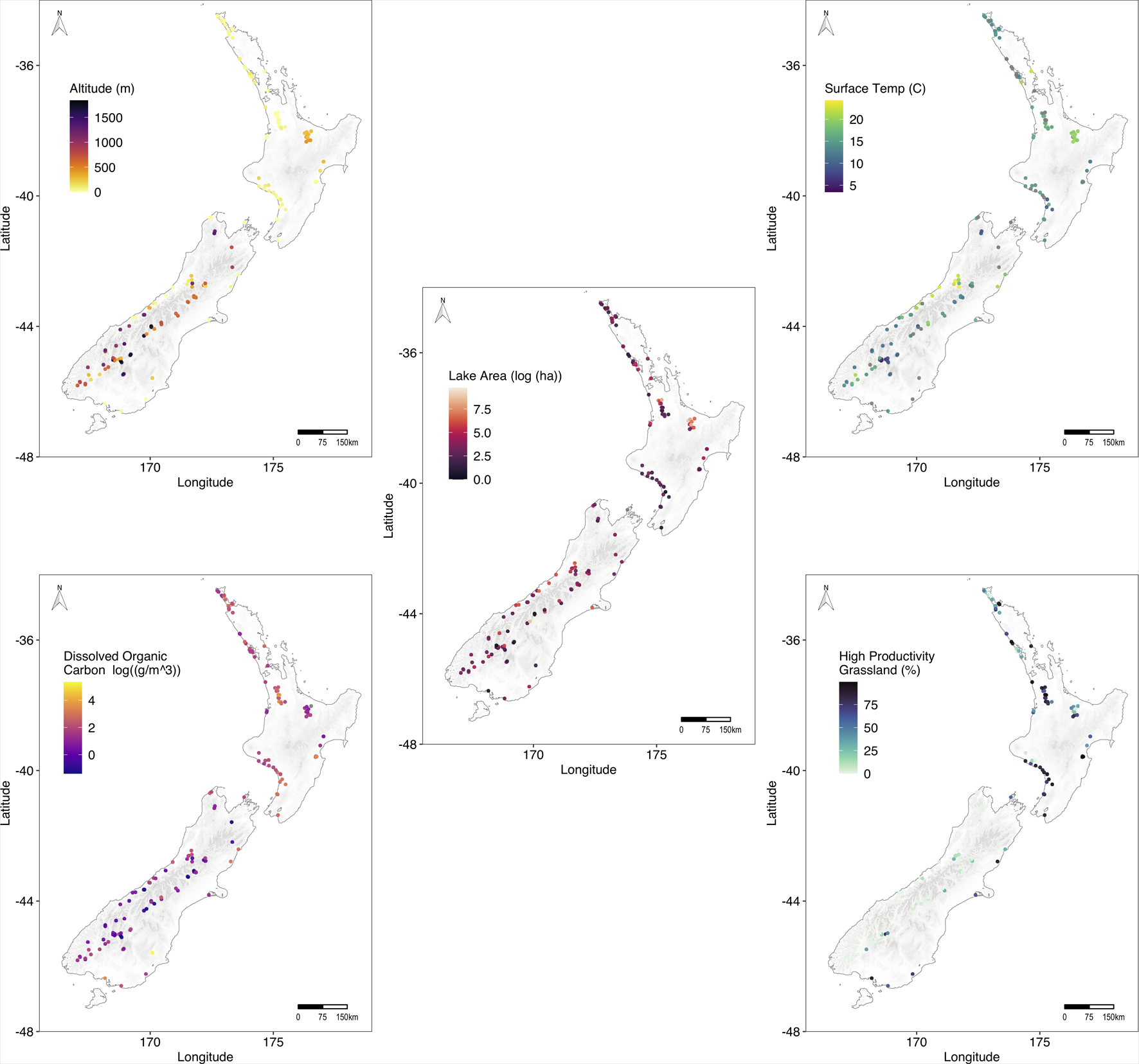

Supplement: Supplementary Figure 1 — Selected environmental and physico-chemical variables and dominant land uses at the study lakes. The environmental data, physico-chemical results, and land use data for all sites are provided in Supplementary Table 1. [file Image_1.TIFF]

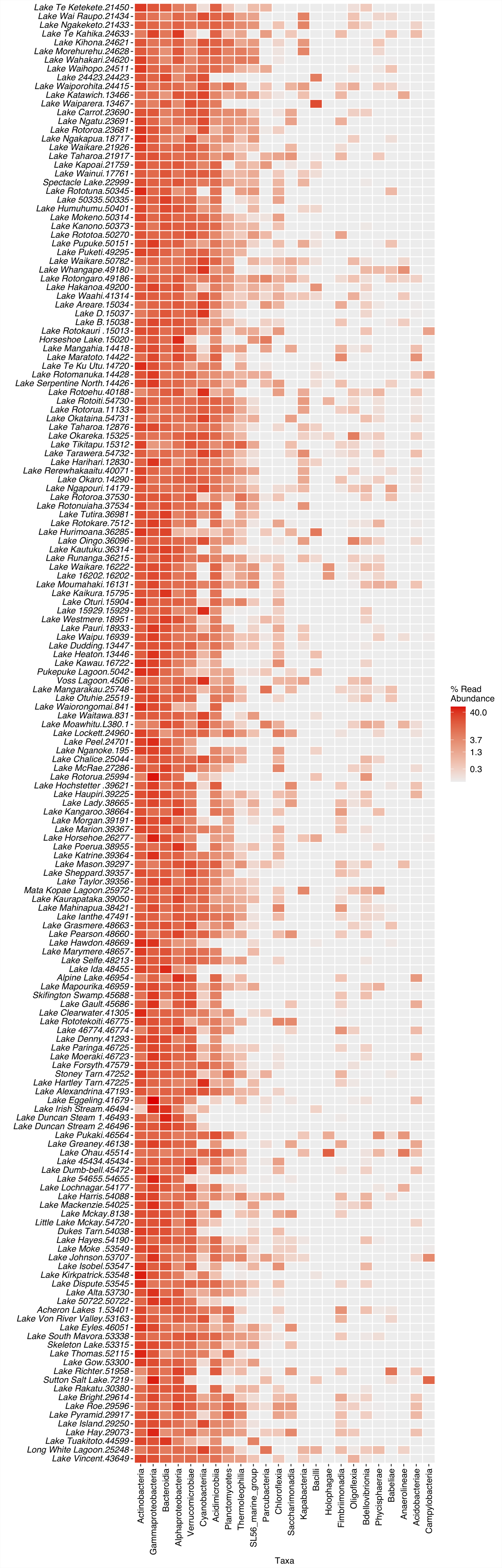

Supplement: Supplementary Figure 2 — Percentage abundance of bacterial classes in 174 New Zealand lakes. Only classes that comprised >0.1% of community are shown. [file Image_2.TIFF]

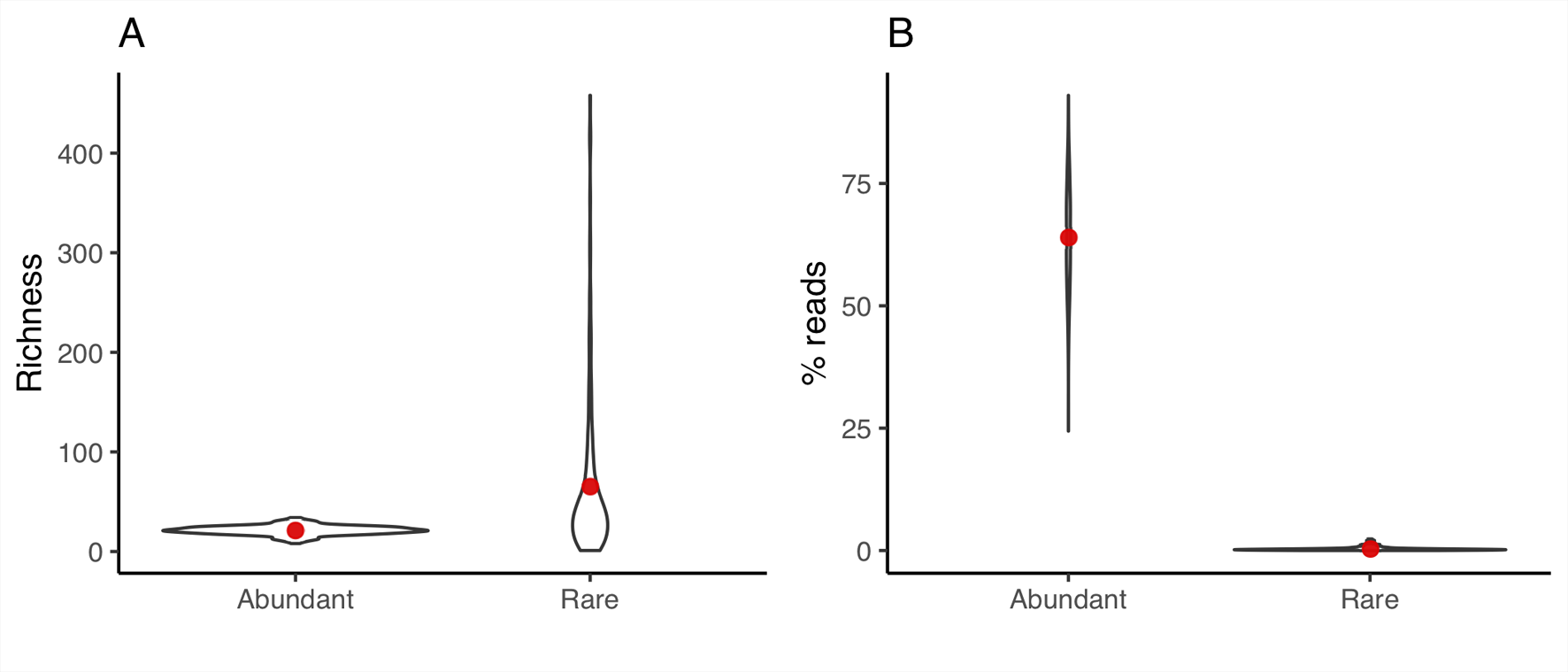

Supplement: Supplementary Figure 3 — Violin plots showing (A) the richness of locally abundant and rare ASVs in a lake and (B) the percentage of reads accounted for by the locally abundant and rare ASVs per lake. [file Image_3.TIFF]

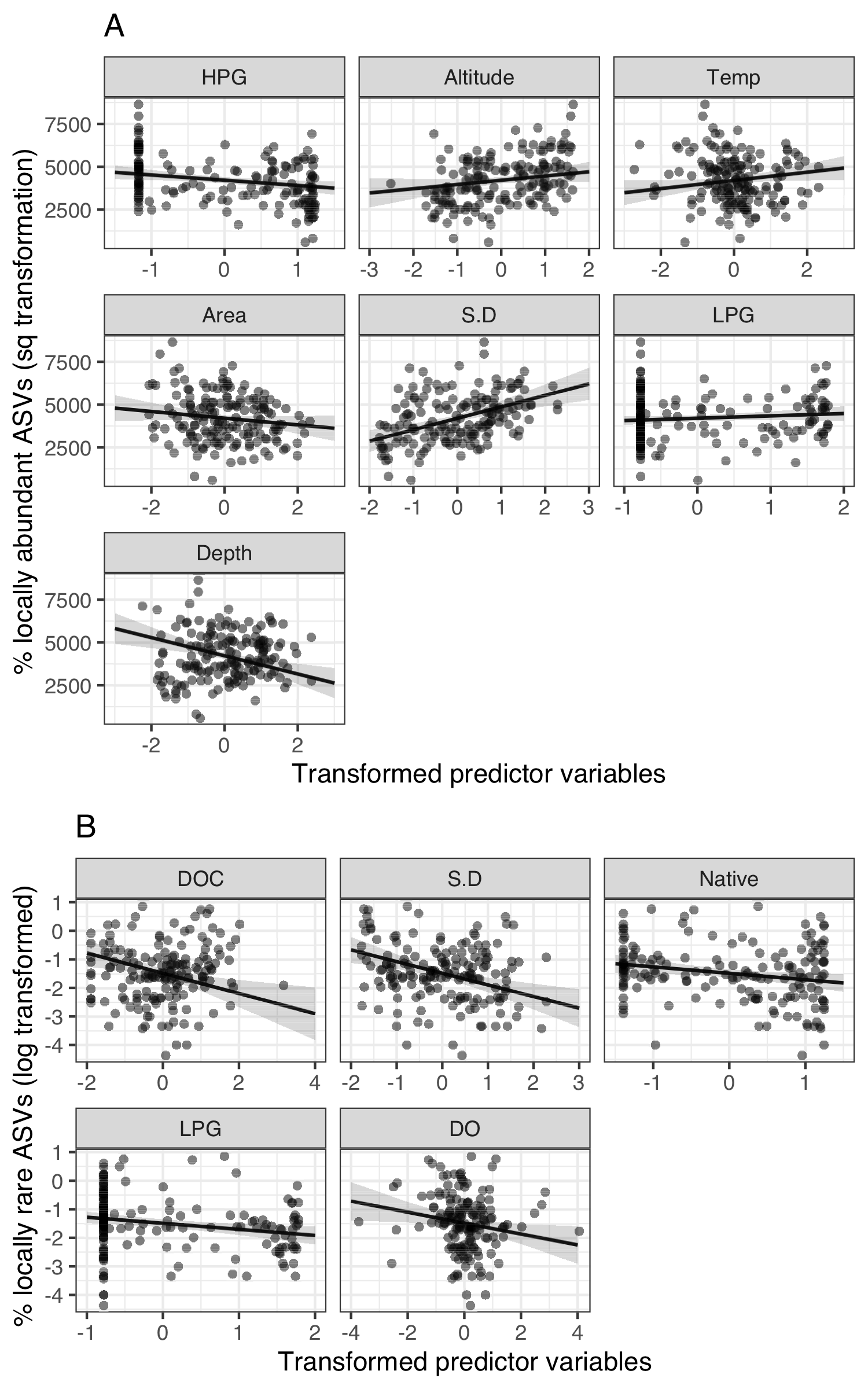

Supplement: Supplementary Figure 4 — Partial effects for the Generalized Linear Model (GLM) for; (A) the proportion of reads attributed to locally abundant amplicon sequence variants (ASVs; >1% of ASVs within a lake) were negatively affected by HPG (p = 0.012) and depth (p < 0.001) while being positively affected by S.D (p < 0.001) and altitude (p = 0.030). The relationship with Temp and LPG was not significant. (B) The proportion of reads attributed to the locally rare ASVs (<0.01% within a lake) with all included environmental variables being negatively associated (p < 0.01). HPG, percentage of highly productive grassland in the catchment; LPG, percentage of low productivity grassland in the catchment; Temp, surface water temperature; S.D, Secchi disk depth; DOC, dissolved organic carbon; DO, dissolved oxygen. [file Image_4.TIFF]
